# Adrenal Washout Under Scrutiny: Spectrum, Pitfalls, and Mimics

**DOI:** 10.7759/cureus.109504

**Published:** 2026-05-23

**Authors:** Surinder Singh, Tripti Jain, Harinder S Chhabra

**Affiliations:** 1 Radiodiagnosis, Gian Sagar Medical College and Hospital, Patiala, IND; 2 Pathology, Gian Sagar Medical College and Hospital, Patiala, IND; 3 Internal Medicine, Adesh Institute of Medical Sciences and Research, Bathinda, IND

**Keywords:** adrenal adenoma, adrenal incidentaloma, adrenal metastasis, adrenal protocol ct, adrenocortical carcinoma, hypervascular metastasis, imaging pitfalls, renal cell carcinoma, washout analysis

## Abstract

Background

Adrenal incidentalomas are frequently encountered on cross-sectional imaging, necessitating accurate non-invasive characterization to guide management. Computed tomography (CT) washout analysis is widely used to distinguish adenomas from non-adenomatous lesions; however, overlap in enhancement characteristics may lead to diagnostic pitfalls, particularly in hypervascular metastases.

Methodology

This retrospective case series includes six patients with incidentally detected adrenal lesions evaluated using a dedicated adrenal protocol on contrast-enhanced CT. Imaging assessment included unenhanced, portal venous, and delayed phase acquisitions, with calculation of the absolute percentage washout (APW). Lesions were characterized based on attenuation, morphology, and enhancement patterns, with correlation with clinical history and histopathological findings where available.

Results

Three lesions demonstrated classic features of lipid-rich adrenal adenomas, including low unenhanced attenuation and rapid washout (APW >60%). Two lesions represented adrenal metastases from biopsy-proven clear cell renal cell carcinoma (RCC), showing avid enhancement and high washout values (APW ~70%), thereby mimicking adenomas. However, these lesions demonstrated enhancement patterns concordant with the primary tumor, with additional metastatic disease in one case. One lesion represented adrenocortical carcinoma, characterized by large size, heterogeneity, necrosis, and intermediate washout.

Conclusion

CT washout analysis, while valuable, should not be interpreted in isolation. Hypervascular metastases, particularly from RCC, may demonstrate adenoma-like washout kinetics, representing an important diagnostic pitfall. Integration of imaging findings with clinical context and enhancement pattern analysis is essential for accurate lesion characterization and appropriate management.

## Introduction

Incidental adrenal lesions (“adrenal incidentalomas”) are increasingly encountered in routine cross-sectional imaging, with a reported prevalence of up to 4%-5% in abdominal computed tomography (CT) studies, rising with age [1]. Accurate characterization of these lesions is essential, as they encompass a wide spectrum ranging from benign adenomas to primary malignancies and metastatic deposits [2]. Imaging plays a central role in non-invasive differentiation, thereby guiding appropriate management and avoiding unnecessary interventions.

CT washout analysis is a widely accepted method for characterizing adrenal lesions, based on the principle that lipid-rich adenomas demonstrate rapid contrast washout compared to non-adenomatous lesions [3]. Absolute percentage washout (APW) ≥60% and relative percentage washout (RPW) ≥40% are commonly used thresholds suggestive of benign adenomas [3,4]. However, these criteria are not infallible, and certain lesions, particularly hypervascular metastases such as those from renal cell carcinoma (RCC), may exhibit washout characteristics overlapping with adenomas, leading to potential diagnostic pitfalls [5,6].

This case series aims to illustrate the imaging spectrum of incidental adrenal lesions encountered in clinical practice, with emphasis on CT adrenal protocol findings. Special focus is placed on the limitations of washout criteria, highlighting cases where hypervascular metastases from RCC mimic benign adenoma kinetics. Through a case-based approach, we aim to reinforce the importance of comprehensive imaging assessment in conjunction with the clinical context.

## Materials and methods

This retrospective case series was conducted at a tertiary care center and included patients with incidentally detected adrenal lesions identified on contrast-enhanced CT (CECT) performed for various clinical indications. Cases demonstrating a spectrum of adrenal pathologies, including benign and malignant etiologies, were selected for illustrative purposes.

All patients underwent dedicated adrenal protocol CT examinations on a multidetector CT scanner. The imaging protocol included unenhanced, portal venous phase, and delayed phase acquisitions (10-15 minutes post-contrast). Attenuation values (in Hounsfield units) were obtained by placing regions of interest (ROIs) within the solid, non-necrotic components of the lesions, avoiding calcifications, hemorrhage, and cystic areas. Care was taken to maintain consistent ROI size and placement across all phases.

Absolute percentage washout (APW) and relative percentage washout (RPW) were calculated using the following standard formulas: $APW = \frac{(E - D)}{(E - U)} \times 100$ and $RPW = \frac{(E - D)}{E} \times 100$, where U represents unenhanced attenuation, E represents enhanced attenuation, and D represents delayed attenuation. Lesions were categorized based on imaging characteristics, including size, attenuation, enhancement pattern, and washout behavior. Correlation with clinical history, particularly the presence of a known primary malignancy, was performed. Additional imaging follow-up or histopathological data, where available, were reviewed to support the diagnosis.

Representative cases demonstrating key imaging features and diagnostic challenges, particularly the overlap between adenomas and hypervascular metastases, were selected for detailed analysis and presentation.

## Results

A total of six patients with incidentally detected adrenal lesions were evaluated. The cases encompassed benign adrenal adenomas, metastatic involvement from renal cell carcinoma, and primary adrenal malignancy, illustrating a spectrum of adrenal pathologies with overlapping imaging characteristics. A summary of the comparison of clinical presentation, imaging findings, and washout characteristics is provided in Table 1. All adenomas demonstrated low unenhanced attenuation and rapid washout characteristics. An additional observation in the present cohort was a relatively consistent D/E ratio pattern, with adenomas demonstrating values of approximately 0.3, whereas metastatic lesions demonstrated values closer to 0.5.

**Table 1:** Clinicoradiological characteristics and CT washout parameters of adrenal lesions across the case series HU = Hounsfield units; APW = absolute percentage washout; RCC = renal cell carcinoma APW was calculated using the following formula: APW = [(E − D)/(E − U)] × 100, where U is unenhanced attenuation, E is enhanced attenuation, and D is delayed attenuation.

Case	Age/Sex	Clinical Condition	Primary Diagnosis	Unenhanced HU	Enhanced HU	Delayed HU	APW (%)	Final Adrenal Diagnosis
1	57/F	Obstructive uropathy with pyonephrosis due to pelvic calculus	Infective obstructive uropathy	11	48	14	92%	Lipid-rich adrenal adenoma
2	27/F	Distal ureteric calculus with grade 1 hydroureteronephrosis	Urolithiasis	6	112	37	71%	Lipid-rich adrenal adenoma
3	55/M	Nonspecific abdominal pain	No significant intra-abdominal pathology	13	98	30	80%	Lipid-rich adrenal adenoma
4	51/M	Biopsy-proven right renal cell carcinoma	Clear cell RCC	34	134	65	69%	Adrenal metastasis
5	62/M	Biopsy-proven left renal cell carcinoma with lung and pancreatic metastases	Disseminated RCC	48	205	94	70%	Adrenal metastasis
6	75/M	Right flank fullness	Large suprarenal mass	42	62	54	40%	Adrenocortical carcinoma

Case 1: Incidental adrenal adenoma in the setting of obstructive uropathy with pyonephrosis

**Figure 1:**
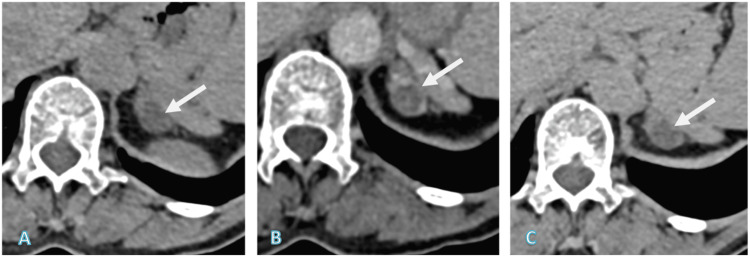
Left adrenal adenoma: adrenal protocol axial CECT image shows a small, well-defined left adrenal lesion (white arrows) with low unenhanced attenuation (A), showing moderate enhancement (B) and rapid contrast washout (C), consistent with lipid-rich adenoma. CECT = contrast-enhanced CT

A 57-year-old woman presented with acute right flank pain, fever, and dysuria, clinically suspected of an infective obstructive uropathy. CECT revealed right renal pyonephrosis secondary to an obstructive pelvic calculus. Incidentally, a well-defined left adrenal lesion measuring 10 × 15 mm was identified. The lesion demonstrated low attenuation on unenhanced CT (11 HU), with enhancement to 48 HU on the portal venous phase and washout to 14 HU on delayed imaging. These imaging characteristics, particularly low baseline attenuation and rapid APW (92%), are diagnostic of a lipid-rich adrenal adenoma (Figure 1). The lesion was unrelated to the presenting clinical condition.

Case 2: Incidental adrenal adenoma in a young patient with a ureteric calculus

**Figure 2:**
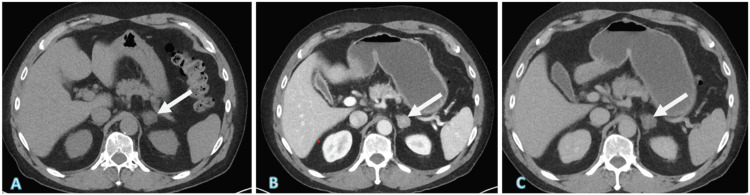
Left adrenal adenoma: axial CECT adrenal protocol images demonstrate a circumscribed left adrenal nodule (white arrows) displaying low attenuation on the non-contrast scan (A), post-contrast enhancement on the venous phase image (B), and significant delayed washout on subsequent imaging (C), characteristic of a lipid-rich adrenal adenoma. CECT = contrast-enhanced CT

A 27-year-old woman presented with radiating left flank pain and dysuria. Imaging revealed a small obstructive calculus in the distal left ureter, causing mild (grade 1) hydroureteronephrosis. An incidental circumscribed left adrenal lesion measuring 15 × 18 mm was noted. The lesion showed attenuation values of 6 HU on unenhanced CT, 112 HU on portal venous phase, and 37 HU on delayed imaging, indicating an APW value of 71%. The low unenhanced attenuation and high washout are characteristic of a benign lipid-rich adrenal adenoma (Figure 2). There were no clinical features suggestive of a functioning adrenal lesion.

Case 3: Incidental adrenal adenoma detected during evaluation of nonspecific abdominal symptoms

**Figure 3:**
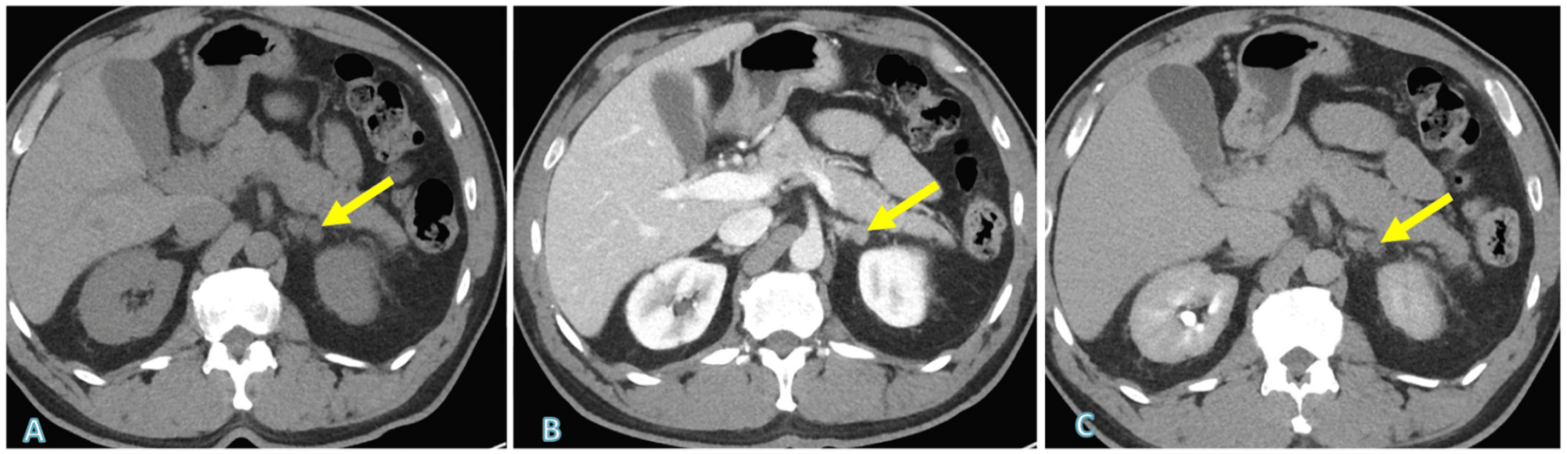
Left adrenal adenoma: axial sections from a dedicated adrenal protocol CT reveal a well-marginated left adrenal nodule (yellow arrows) with low baseline density on unenhanced imaging (A), interval enhancement following contrast administration (B), and marked washout on delayed phase images (C), in keeping with adrenal adenoma.

A 55-year-old man underwent CECT for evaluation of nonspecific abdominal discomfort. No significant intra-abdominal pathology explaining the symptoms was identified. However, a small, well-circumscribed left adrenal lesion measuring 7 × 8 mm was incidentally detected. The lesion demonstrated low attenuation (13 HU) on unenhanced CT, with enhancement to 98 HU and subsequent washout to 30 HU (80%) on delayed imaging. These findings are consistent with a lipid-rich adrenal adenoma (Figure 3). The lesion was considered incidental and clinically non-contributory.

Case 4: Adrenal metastasis from clear cell RCC mimicking adenoma on washout analysis

**Figure 4:**
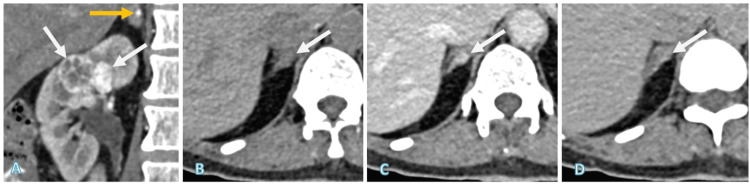
RCC metastasis – right adrenal: coronal CECT (A) shows an irregular hypervascular right renal mass (white arrows) with a synchronous right adrenal lesion (yellow arrow) showing similar hyperenhancement. Axial CECT shows a right adrenal nodule with high unenhanced attenuation (B) and avid enhancement (C), demonstrating rapid washout (D) mimicking adenoma; however, enhancement parallels known RCC, favoring metastasis. RCC = renal cell carcinoma; CECT = contrast-enhanced CT

A 51-year-old man with biopsy-proven clear cell RCC of the right kidney underwent staging evaluation. Imaging revealed a small right adrenal nodule measuring 5 × 6 mm. The lesion demonstrated relatively high unenhanced attenuation (34 HU) and marked enhancement (134 HU) on the portal venous phase, with delayed attenuation of 65 HU. Despite an APW value of 69%, which falls within the range typically suggestive of adenoma, the lesion was interpreted as metastatic based on the clinical context of known RCC and the hypervascular enhancement pattern mirroring the primary tumor. This case highlights the limitation of washout criteria in oncologic settings (Figure 4).

Case 5: Multifocal metastatic disease, including adrenal involvement from clear cell RCC

**Figure 5:**
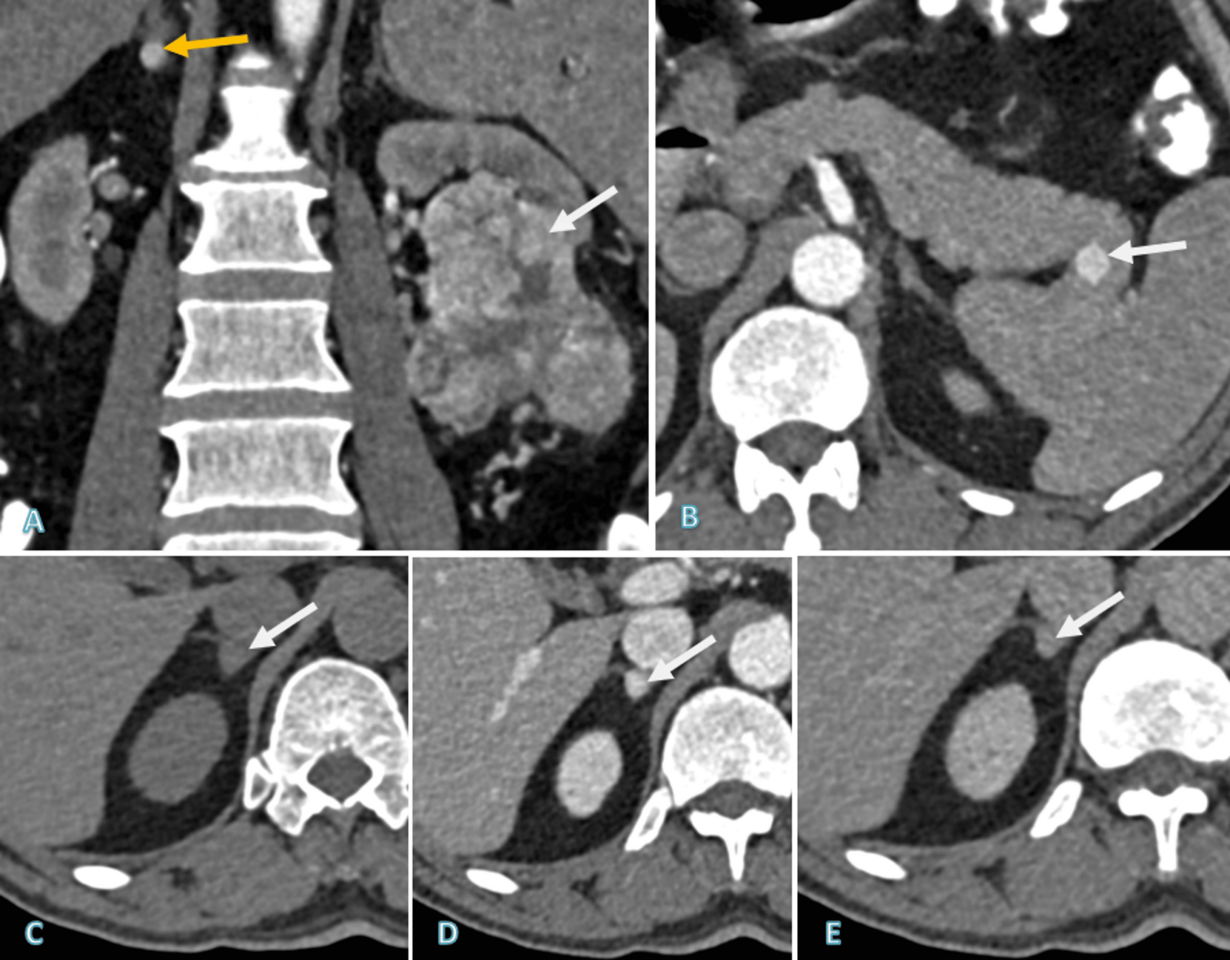
Left RCC metastasis – right adrenal with dissemination: coronal CECT (A) shows an irregular hyperenhancing left renal mass (white arrow) and a right adrenal nodule (yellow arrow) mimicking its enhancement. Axial CECT (B) reveals a synchronous, hypervascular metastatic nodule in the tail of the pancreas (white arrow). Axial CECT images show a right adrenal nodule (white arrow) with high unenhanced attenuation (C) and avid enhancement (D), demonstrating rapid washout (E) mimicking adenoma; however, enhancement parallels primary RCC, consistent with metastatic disease. RCC = renal cell carcinoma; CECT = contrast-enhanced CT

A 62-year-old man with biopsy-proven clear cell RCC of the left kidney underwent staging imaging. A right adrenal lesion measuring 6 × 8 mm was identified, demonstrating high unenhanced attenuation (48 HU), intense enhancement (205 HU), and delayed attenuation of 94 HU. Although the calculated APW was 70%, suggestive of adenoma, the lesion was consistent with metastasis in view of the known primary malignancy. Additional metastatic lesions were identified in the left lower lobe of the lung and the pancreatic tail, all demonstrating similar hypervascular enhancement characteristics. The constellation of findings confirmed disseminated metastatic disease with adrenal involvement (Figure 5).

Case 6: Large adrenocortical carcinoma presenting as a heterogeneous suprarenal mass

**Figure 6:**
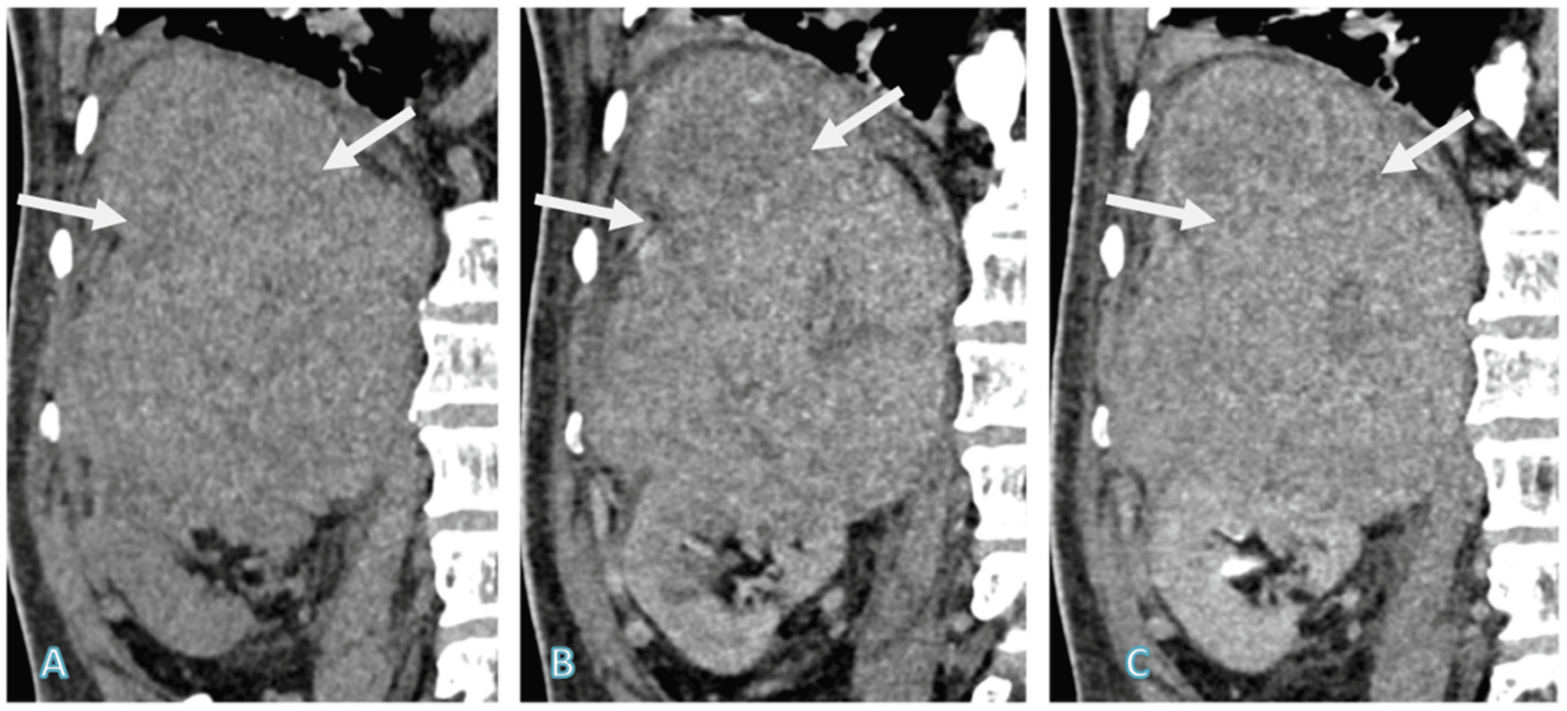
Adrenocortical carcinoma: coronal CECT shows a large heterogeneous right adrenal mass (white arrows) with high unenhanced attenuation (A), irregular margins, necrosis, mild non-uniform enhancement (B) and suboptimal washout (C), consistent with adrenocortical carcinoma. CECT = contrast-enhanced CT

A 75-year-old man presented with right flank fullness without other significant symptoms. CECT revealed a large (15 × 17 cm), lobulated, solid mass in the right suprarenal region, causing inferior displacement of the right kidney. The lesion demonstrated heterogeneous enhancement with areas of internal necrosis. Attenuation values were 42 HU on unenhanced CT, 62 HU on portal venous phase, and 54 HU on delayed imaging, indicating low washout (70%). The large size, heterogeneity, necrosis, and suboptimal washout were highly suggestive of a primary adrenal malignancy (Figure 6). Histopathological examination confirmed adrenocortical carcinoma.

## Discussion

Adrenal incidentalomas are increasingly detected due to the widespread use of cross-sectional imaging, with reported prevalence ranging from 1% to 5% in general CT series [1,2]. Accurate characterization is essential, as management differs significantly between benign adenomas and malignant or metastatic lesions. CT, particularly multiphasic contrast-enhanced protocols, remains the cornerstone for noninvasive evaluation [3,4].

CT washout analysis is one of the most widely validated techniques for differentiating adrenal adenomas from non-adenomatous lesions. Lipid-rich adenomas typically demonstrate low unenhanced attenuation (<10-15 HU) and rapid contrast washout, with absolute percentage washout ≥60% and relative percentage washout ≥40% considered highly specific diagnostic thresholds. These criteria have been consistently supported across multiple studies, with reported specificity exceeding 90% in appropriately selected lesions [3,4].

In our series, all three adenomas (Cases 1, 2, and 3) demonstrated classic imaging features, including low baseline attenuation and rapid washout kinetics. These findings are concordant with the established literature and reinforce the reliability and reproducibility of CT washout analysis in typical lipid-rich adenomas [3,4,7,8]. Interestingly, the D/E ratio also demonstrated a relatively consistent separation between adenomas and metastatic lesions in our cohort, with lower values observed in adenomas compared to metastases. Although limited by the small sample size, this observation may reflect differing enhancement-washout kinetics and could represent a potentially useful adjunctive imaging parameter warranting further evaluation in larger studies. Collectively, these findings highlight the continued clinical utility of washout protocols as a first-line diagnostic tool in routine radiology practice.

However, an important diagnostic limitation is illustrated by Cases 4 and 5. Despite demonstrating high APW values (>60%), both lesions were proven adrenal metastases from clear cell RCC. RCC is a characteristically hypervascular tumor, and its metastases frequently exhibit avid arterial enhancement followed by relatively rapid washout, thereby mimicking the enhancement kinetics of adrenal adenomas [5,6]. This overlap represents a recognized diagnostic pitfall and reduces the specificity of washout criteria in patients with known hypervascular primary malignancies.

Several studies have emphasized that washout thresholds, although highly specific in general populations, may be less reliable in oncologic settings [5,6]. In such cases, imaging interpretation must extend beyond quantitative washout values. Integration of clinical history (particularly known primary malignancy), comparison of enhancement patterns with the primary tumor, lesion morphology, and the presence of synchronous metastatic disease becomes critical. In Case 5, the presence of additional metastatic lesions with identical enhancement characteristics provided a key diagnostic clue favoring metastatic disease over a benign adenoma. Importantly, our findings provide practical evidence that hypervascular metastases can satisfy established washout thresholds, reinforcing that these criteria may be insufficient when used in isolation in oncologic patients. This observation adds to the existing literature by illustrating real-world diagnostic overlap and underscores the need for a more integrated interpretative approach.

The case of adrenocortical carcinoma (Case 6) demonstrated imaging features typical of a primary malignant adrenal tumor, including large size, heterogeneous enhancement, necrosis, and relatively low washout values [9]. These morphological characteristics remain important discriminators, particularly when quantitative washout parameters are indeterminate or potentially misleading.

Limitations

This study has certain limitations. First, the sample size is small, reflecting the nature of a case series, which limits generalizability. Second, selection bias may be present, as cases with atypical or discordant imaging features are more likely to be reported. Third, not all lesions may have uniform imaging protocols or timing, which can influence washout calculations. Additionally, arterial phase imaging data were not consistently available, precluding assessment of wash-in enhancement characteristics of the adrenal lesions. Future studies incorporating multiphasic enhancement analysis may provide further insights into lesion characterization. Finally, histopathological confirmation was available only in selected cases, which may introduce verification bias. Despite these limitations, the cases effectively highlight a clinically relevant diagnostic pitfall.

Overall, this series underscores that while CT washout analysis is a robust and widely used tool, it should not be interpreted in isolation. Hypervascular metastases, particularly from RCC, can demonstrate misleading washout characteristics, necessitating careful correlation with clinical and imaging context to avoid diagnostic errors.

## Conclusions

CT washout analysis remains a highly effective and practical method for characterizing adrenal lesions in routine clinical practice. When classic imaging criteria are present, it provides a confident, noninvasive diagnosis of adrenal adenoma and can help avoid unnecessary interventions. However, this study highlights an important diagnostic caveat: not all lesions that meet washout thresholds are benign. Hypervascular metastases, especially from renal cell carcinoma, can closely mimic adenomas on washout imaging. This reinforces the need for radiologists to interpret washout findings within the broader clinical and imaging context rather than relying solely on numerical thresholds.

A thoughtful, integrated approach that considers patient history, lesion morphology, enhancement patterns, and associated findings is essential for accurate diagnosis. Recognizing these potential overlaps can significantly improve diagnostic confidence and help guide appropriate patient management.
